# Prevalence and Factors Associated with Infections After Acute Ischemic Stroke: A Single-Center Retrospective Study over Five Years

**DOI:** 10.3390/epidemiologia6030046

**Published:** 2025-08-11

**Authors:** Weny Rinawati, Aryati Aryati, Abdulloh Machin, Stefan Kiechl, Gregor Broessner

**Affiliations:** 1 Doctoral Program in Medical Science, Faculty of Medicine, Universitas Airlangga, Surabaya 60132, Indonesia; weny.rinawati-2022@fk.unair.ac.id; 2 Laboratory and Blood Bank Unit, National Brain Center Hospital Mahar Mardjono, Jakarta 13630, Indonesia; 3 Department of Clinical Pathology, Faculty of Medicine, Universitas Airlangga, Surabaya 60132, Indonesia; 4 Dr. Soetomo General Academic Hospital, Surabaya 60132, Indonesia; 5 Department of Neurology, Faculty of Medicine, Universitas Airlangga, Surabaya 60132, Indonesia; 6 Airlangga University Hospital, Surabaya 60115, Indonesia; 7 Department of Neurology, Medical University of Innsbruck, 6020 Innsbruck, Austria; stefan.kiechl@i-med.ac.at (S.K.); gregor.broessner@i-med.ac.at (G.B.); 8 VASCage—Center for Clinical Stroke Research, 6020 Innsbruck, Austria

**Keywords:** infection, ischemic stroke, microbiologic culture, post-stroke infection, prevalence

## Abstract

Background/Objectives: Infections after stroke are a serious medical problem and have a significant impact on the outcome of stroke, but data regarding the Asian population are limited. This study aims to determine the bacterial and fungal profile of pathogenic organisms of infections after acute ischemic stroke (AIS). Methods: This is a retrospective study using the medical records of patients at least 18 years old who were hospitalized with AIS in a tertiary stroke hospital from 1 January 2018 to 31 December 2022. Demographic, patient-related, and other examination data were extracted from hospital medical records. Infections after AIS were defined as any infection that developed during the acute phase of ischemic stroke and was confirmed by microbiologic culture as the gold standard. Factors associated with infection were analyzed using multiple logistic regression. Results: Among 599 AIS patients with infection who underwent microbiologic culture, the prevalence of infection with an isolated pathogen was 21.4%, and most organisms were from sputum. Positive microbiologic culture revealed that bacteria such as *K. pneumoniae*, *E. coli*, *A. baumannii*, and *S. aureus* were the most common causes of infection, while fungi were rare. During the COVID-19 period, bacteria developed resistance to antimicrobials, including β-lactamase antibiotics for Gram-negative bacteria and methicillin for Gram-positive bacteria. Care in the intensive ward, including the stroke unit, reduced the risk of a positive microbiological culture in the COVID-19 and non-COVID-19 period. Urinary catheters promoted infections in the non-COVID-19 period, whereas steroids, total parenteral nutrition, and tracheostomy were negatively associated with infections after AIS in the COVID-19 period. Conclusions: The prevalence and factors associated with infection after stroke changed during the COVID-19 period. The risk of infection after stroke requires preventive measures such as early dysphagia screening.

## 1. Introduction

The World Stroke Organization (WSO) 2025 stroke burden estimates revealed that stroke remains the second leading cause of death (about 7 million) and the third leading cause of death and disability globally, with over 160 million years of healthy life lost each year due to stroke-related death and disability. Ischemic stroke incidence constitutes 65% of all incident strokes and over 7.8 million new cases each year. Almost 70 million people who are alive have experienced ischemic stroke [1]. Ischemic stroke is a stroke caused by sudden arterial occlusion due to a thrombus that forms directly in the area of occlusion (thrombotic ischemic stroke) or that forms in another area of the circulation, causing arterial obstruction (embolic ischemic stroke). The care of ischemic stroke requires integrated and comprehensive care, including the prevention of complications that can affect stroke outcomes, with the largest service costs based on data from the World Health Organization (WHO) over the past two decades [1]. Annually, 3.6 million people die from ischemic stroke, with the estimated global cost of stroke being over USD 890 billion per year, which is projected to almost double by 2050 [2].

Complications that are often encountered in the care of acute stroke patients include infections, among others, which need to be addressed, considering that this incident affects the high cost of stroke services [3]. A previous study indicated that infectious complications were independently associated with prolonged hospital stay (OR = 1.78) [4]. Even though the prevalence of infection after stroke decreased, which was reported from 30% to 9.14% (8.23–10.10%), the mortality remained at 15.91% [5]. Therefore, one crucial part of the early management of stroke patients is the prevention of infection after stroke [4].

Most infections after stroke occur in the first three to seven days after the stroke in patients who experience complications during treatment or up to the seventh day in 15% of acute ischemic stroke (AIS) patients [5]. Previous research has shown that age, diabetes mellitus, disturbance of consciousness, the National Institutes of Health Stroke Scale (NIHSS) score at admission, invasive surgery, and chronic obstructive pulmonary disease (COPD) were risk factors for infections after AIS [6].

The presence of these various factors is a challenge to the management of infection as a complication after AIS. Alternatives for preventing infections after stroke that have been studied include the administration of immunomodulators in experimental animals and preventive antibiotics, which have produced varying outcomes. Since the use of antibiotics might be non-optimal, prophylactic antibiotics did not always improve outcomes or reduce the overall incidence of infection after stroke [7]. In addition, being mainly unmanaged and relying on antibiotics for treatment would lead to the development of antibiotic resistance [8].

Relying on antibiotics for treatment occurred during the coronavirus disease 2019 (COVID-19) pandemic. Healthcare has transformed to involve the abundant use of antibiotics, increasing by more than 75%. Though antibiotics do not cure COVID-19, the overuse of antibiotics for treating COVID-19 patients was out of concern that bacterial coinfection would occur as a result of panic about an unknown disease, which has similar symptoms to pneumonia and a higher death rate [8,9]. A meta-analysis study showed that the COVID-19 pandemic has had an impact on the accelerated emergence and transmission of antibiotic resistance across healthcare settings [8].

The aim of this study was to determine whether the profile of pathogenic agents of infections after AIS was also influenced by the COVID-19 pandemic period, during which antibiotics were used generously. This study, over five years, was expected to result in a dynamic profile of pathogenic agents of infections after AIS, for which there were limited studies.

## 2. Materials and Methods

### 2.1. Study Design and Participants

This retrospective cross-sectional study was performed in the National Brain Center Hospital (NBC) Mahar Mardjono in Jakarta, Indonesia, which served as the study center. The NBC is a public tertiary neurology teaching and referral center that provides a comprehensive range of inpatient services, including intensive care and stroke units. This study was approved by the Research Ethics Committee, and the need for informed consent, being non-interventional, was waived.

**Figure 1:**
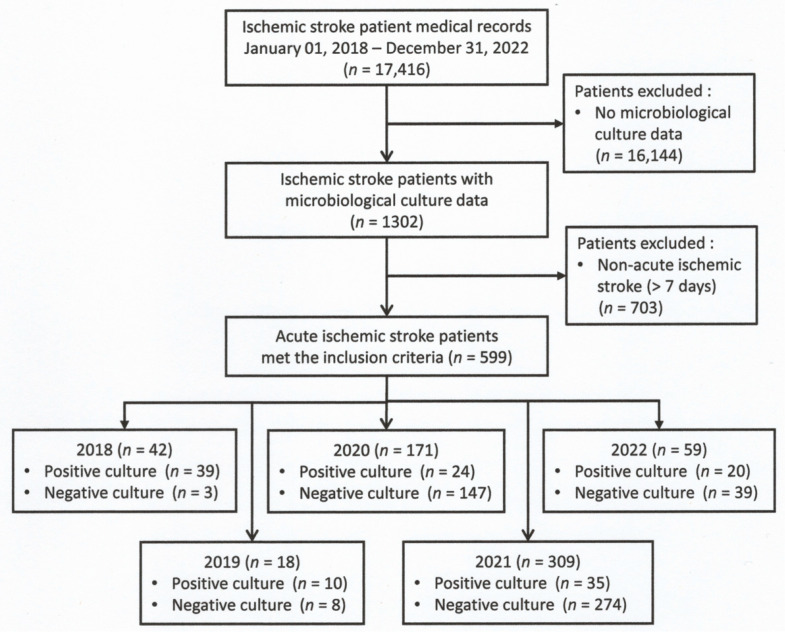
Flowchart of the study participants.

In this study, the data were collected through a comprehensive review of hospital medical records. The study period was from 1 January 2018 to 31 December 2022 (Figure 1). All patients at least 18 years of age with a diagnosis of AIS within a week after stroke onset who had microbiology cultures were included in this study [10].

Acute ischemic stroke was diagnosed by reliable clinical symptoms within a week after the onset of stroke or confirmed by radiological examination (computed tomography scan or magnetic resonance imaging [11]). Infection was identified by reliable clinical symptoms or diagnostic guidelines of the disease, including pneumonia [12,13,14], urinary tract infection (UTI) [12,15], other infections [16], or sepsis [16,17], and confirmed by microbiological culture as the gold standard. Microbiological culture was performed from samples of body fluids and infected tissue according to the standard methods [18]. A positive culture was determined if microorganisms grew in the sample culture; otherwise, it was defined as negative.

Demographic data (age and sex), patient-related factors, and other investigations were obtained from medical records. Patient-related factors potentially associated with infection included clinical factors (e.g., diabetes mellitus (DM) and human immunodeficiency virus (HIV)) and iatrogenic factors (e.g., use of a ventilator, central venous catheter (CVC), nasogastric tube (NGT), and urinary catheter). We also considered COVID-19 as a concomitant disease, leucocyte count (more than 10,000/µL was leukocytosis and less than 5000/µL was leukopenia) [11], treatments such as antibiotics, steroids, total parenteral nutrition (TPN), and transfusions, and procedures (e.g., tracheostomy, digital subtraction angiography (DSA), and head surgery (e.g., craniectomy or craniotomy). In identifying factors associated with infection in AIS, this study was divided into the non-COVID-19 period (2018, 2019, and 2022) and the COVID-19 period (2020–2021), as in Indonesia, COVID-19 emerged in 2020.

### 2.2. Data Analysis

The research subjects’ characteristics were provided descriptively. Using the Kolmogorov–Smirnov test of normality data distribution, the normal distribution of continuous variables was reported as the mean with standard deviation (SD); otherwise, it was reported as the median with interquartile range (IQR, percentiles 25 and 75). Furthermore, the categorical data were expressed in numbers (*n*) and percentages (%). Analysis with the χ^2^ test was used to determine the differences in the categorical variable between the two groups. The *p*-values were derived from two-sided tests, and *p*-values less than 0.05 were considered statistically significant.

To identify factors associated with the positivity rate of microbial growth, variables with a *p*-value less than 0.25 from the univariate analysis were included in the multivariate logistic regression analysis [19]. Factors that influence infections after AIS were obtained and analyzed using the multivariate logistic regression analysis. In a univariate analysis, *p*-values less than 0.25 were accepted for inclusion in the initial model of multivariate logistic regression analysis to discover the characteristics linked to the positive rate of bacterial growth. To ensure the simultaneous consideration of multiple confounding variables, the multivariate logistic regression model was utilized. Along the entire modeling procedure, both primary and confounding variables were included. Based on their significance to the study, interaction variables were eliminated one at a time if the odds ratio for the main variable changed by more than 10%; confounding was proposed. The *p*-values less than 0.05 were considered statistically significant in the final model. The Statistical Package for the Social Sciences (IBM^®^ SPSS^®^ Statistics, New York, NY, USA) version 26 was used for our analyses.

## 3. Results

As part of this study, 17,416 patients who had an ischemic stroke in the last five years (2018–2022) were thoroughly examined on the basis of their medical records during their hospital stay. Of these patients, 1302 patients with infection after stroke underwent sample culture. We excluded 703 patients with non-AIS (more than 7 days), leaving 599 patients with infections after AIS underwent microbiological culture, including 42, 18, and 59 patients from the non-COVID-19 period (2018, 2019, and 2022) and 171 and 309 in the COVID-19 period (2020 and 2021), respectively (Figure 1).

### 3.1. Demographic Data

The mean age was 60.0 ± 5.0 years, with a male predominance (*n* = 407, 67.9%), which was constant over the five years. Around 70% of AIS patients did not stay in the intensive/stroke unit. The most prevalent comorbidities in AIS patients were COVID-19 (51.8%), followed by DM (27.2%). Leukopenia was observed in 392 (65.4%) patients. The common medical devices used were CVC (*n* = 179, 29.9%) and a ventilator (*n* = 61, 10.2%), whilst treatments and procedures were antibiotics and tracheostomy, at 61.3% and 6.5%, respectively. A summary of baseline characteristics is shown in Table 1.

Clinical manifestations of infection in this study were fever (*n* = 119, 19.9%), sepsis (*n* = 223, 37.2%), and pneumonia (*n* = 238, 39.7%), while meningitis or encephalitis, infected wounds, and urinary tract infections were less common. Compared to the non-COVID-19 period, there was an increased prevalence of fever (32.1 vs. 6.7%, *p* < 0.001), sepsis (39.4 vs. 28.6%, *p* < 0.001), and pneumonia (41.5 vs. 32.8%, *p* < 0.001) during the COVID-19 period (Table 2).

### 3.2. Prevalence and Microbiological Data

**Figure 2:**
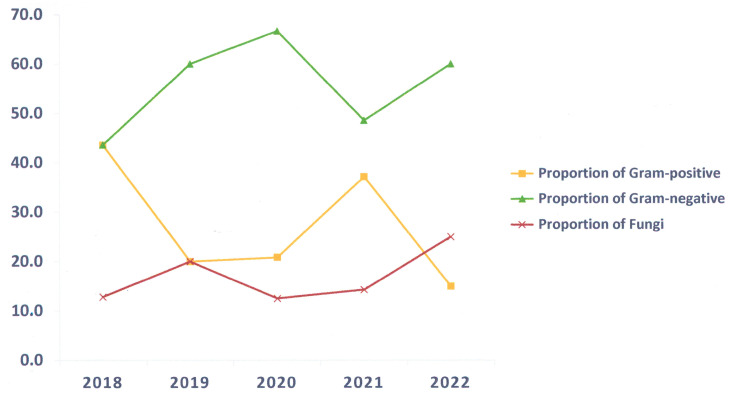
Percentage of different types of infections.

In 599 AIS patients with microbiological cultures, pathogens were detected in 21.4%. The proportion of Gram-positive bacteria decreased during the non-COVID-19 period and increased during the COVID-19 period. The proportion of Gram-negative bacteria increased before and during the first year of the COVID-19 period and decreased during the second year of the COVID-19 period, followed by an increase after the COVID-19 period. The proportion of fungi decreased during the COVID-19 period and increased during the non-COVID-19 period (Figure 2).

The number of samples has increased significantly during the COVID-19 period. The most common samples in the five-year period were blood (70.6%) and sputum (18.5%). The majority of organisms were isolated from sputum (90.1%), pus (63.6%), and urine (29.2%) (Table 3).

Gram-negative bacteria predominated in the growth of pathogenic organisms over five years (53.1%), followed by Gram-positive bacteria (31.3%) and fungi (15.6%). The proportion of fungi obtained from microbiological cultures was 15.6% (Table 4).

Compared to the non-COVID-19 period, there was a decreased proportion of all pathogens, which were Gram-negative bacteria (6.9 vs. 11.4%, *p* < 0.001), Gram-positive bacteria (3.8 vs. 18.5%, *p* < 0.001), and fungi (1.7 vs. 10.1%, *p* < 0.001) during the COVID-19 period (Table 5).

**Figure 3:**
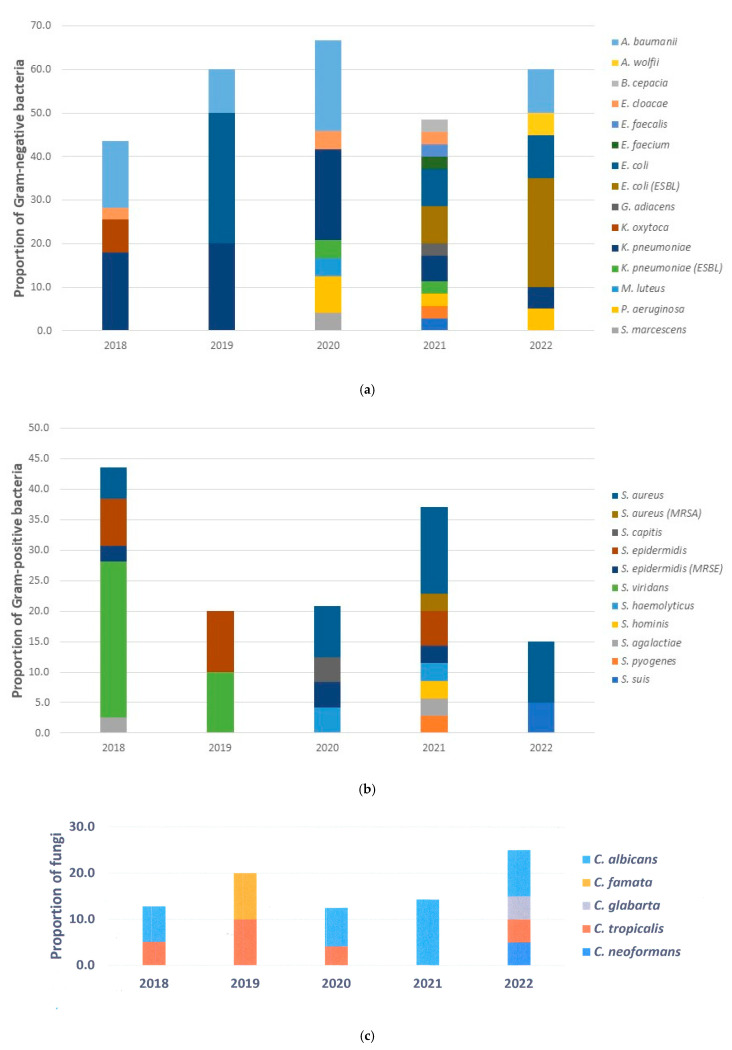
(**a**) Distribution of Gram-negative bacteria. (**b**) Distribution of Gram-positive bacteria. (**c**) Distribution of fungi.

The three most common Gram-negative bacteria were *Klebsiella pneumoniae* (27.9%), *Escherichia coli* (23.5%), and *Acinetobacter baumannii* (20.6%). Two of the 17 *K. pneumoniae* bacteria during the COVID-19 period were ESBL (extended-spectrum beta-lactamases) strains. *E. coli* increased during the COVID-19 period, and half of the *E. coli* were ESBL strains (8 out of 16). In contrast, *A. baumannii* was not detected throughout the second year of the COVID-19 period, although it was the second pathogen after *K. pneumoniae* in the early non-COVID-19 period (Figure 3a).

Among Gram-positive bacteria, *Staphylococcus aureus* (30.0%), *Streptococcus viridans* (27.5%), and *Staphylococcus epidermidis* (22.5%) were the predominant bacteria. One in twelve *S. aureus* were methicillin-resistant (MRSA) strains that emerged during the second year of the COVID-19 period, while methicillin-resistant *S. epidermidis* (MRSE) were found in three out of six. *S. viridans* (26.5%). These were the most ecumenical bacteria that emerged in the early non-COVID-19 period and were no longer found during the COVID-19 period. After the COVID-19 period, only *S. aureus* and *Streptococcus suis* were detected. In this study, a widespread range of bacteria was detected in the second year of the COVID-19 period (Figure 3b).

*Candida albicans* was the predominant fungus both in the non-COVID-19 and COVID-19 periods. However, *C. albicans* did not grow in the second year of the non-COVID-19 period, whereas in the second year of the COVID-19 period, it was the only pathogen that grew in the microbiological culture. Besides *Candida* spp., *Cryptococcus neoformans* was also found in the microbiological culture (Figure 3c). Regarding the patient with Cryptococcosis, this study only found one male patient in the non-COVID-19 pandemic period (in 2022). The patient, aged 41 years, was in the regular ward, had signs of meningitis, and a leukocyte count of 5200/µL. The results of the HIV testing strategy using three consecutive tests were reactive. There were no other comorbidities such as DM or COVID-19. The patient used medical devices such as a catheter and an NGT, without a history of prolonged corticosteroid therapy.

### 3.3. Factors Associated with Infections After AIS

The factors associated with the positivity rate of microbiological culture in AIS are shown in Table 6, including all variables with a *p* < 0.25, which were age ≥ 60 years, intensive/stroke unit care, HIV, COVID-19, leukocytosis, ventilator, CVC, NGT, use of antibiotics, steroid, TPN, transfusion, tracheostomy, DSA, and head surgery (Supplementary Table S1). In the non-COVID-19 period, the factors associated with the positivity rate of microbiological culture were intensive/stroke unit care, DM, leukocytosis, leukopenia, ventilator, CVC, urinary catheter, antibiotic, steroids, and DSA (Supplementary Table S2). In addition, in the COVID-19 period, the factors associated with the positivity rate of microbiological culture were an age over 60 years, stay in the intensive care unit, HIV, COVID-19, leukocytosis, leukopenia, ventilator, CVC, NGT, urinary catheter, steroid, TPN, transfusion, tracheostomy, and head surgery (Supplementary Table S3).

The variables associated with a risk of bacterial infection were intensive/stroke unit care stay (OR 0.23; 95% CI 0.14–0.374, *p* < 0.001), COVID-19 (OR 4.66; 95% CI 2.69–8.08, *p* < 0.001), NGT (OR 0.46; 95% CI 10.23–0.92, *p* = 0.029), use of steroids (OR 0.23; 95% CI 0.12–0.47, *p* < 0.001), TPN (OR 0.15; 95% CI 0.06–0.37, *p* < 0.001), and DSA (OR 0.22; 95% CI 0.05–1.00, *p* = 0.050). In the non-COVID-19 period, with a *p*-value < 0.05, the variables were intensive/stroke unit care stay (OR 0.35; 95% CI 0.14–0.89, *p* < 0.001), leukocytosis (OR 5.51; 95% CI 1.80–16.91, *p* = 0.003), urinary catheter (OR 13.12; 95% CI 1.68–102.1, *p* = 0.014), and use of antibiotics (OR 0.15; 95% CI 0.06–0.37, *p* < 0.001). In the COVID-19 period, the variables associated with a risk of bacterial infection with a *p*-value < 0.05 were intensive/stroke unit care (OR 0.17; 95% CI 0.09–0.33, *p* < 0.001), leukocytosis (OR 0.35; 95% CI 0.14–0.89, *p* < 0.001), use of steroids (OR 0.33; 95% CI 0.13–0.87, *p* = 0.024), TPN (OR 0.21; 95% CI 0.07–0.57, *p* = 0.002), and tracheostomy (OR 0.20; 95% CI 0.08–0.51, *p* = 0.001) (Table 7).

## 4. Discussion

Microbiologic cultures were frequently positive during the non-COVID-19 period (33–92%) and decreased to 11–14% during the COVID-19 period. Previous studies revealed that during the COVID-19 pandemic, the proportion of patients with bacterial or secondary infections was relatively low [20]. In addition, microbiologic culture was commonly used to determine whether the patient had a secondary infection. The prevalence in this study was 21.4%, lower than in previous studies, since the prevalence was not based only on clinical symptoms but also on culture positivity, which is the gold standard for infection diagnosis.

Sputum samples were the most common microbiological culture samples in five years, denoting that pneumonia was the most common clinical manifestation of infections after AIS. Previous studies in intensive care and general wards revealed that pneumonia is mainly diagnosed in the first days after stroke [21]. The pneumonia in this study was higher compared to the pneumonia infection rate in most previous studies, which ranged from 1% to 33%. It is believed that pneumonia in stroke patients can occur as a result of dysphagia, which leads to pulmonary aspiration within the first 72 h. This condition can significantly increase the risk of mortality [22]. Therefore, the use of NGT in this study was associated with a decrease in microbiology culture positivity rates of 46% in AIS during the five years.

The most prevalent cause of infection in this study was bacteria. Most pathogens were Gram-negative bacteria such as *K. pneumoniae*, *E. coli*, and *A. baumannii*, while *S. aureus* was the most common cause of Gram-positive bacteria. These pathogens are mostly nosocomial infections, which is consistent with previous studies showing that infections caused by Gram-negative bacteria and *S. aureus* are frequently observed in hospitals. *Streptococcus* species were also identified in our study, with *S. viridans* being a predominant species. Nowadays, *Streptococcus* species remain the most ecumenically identified pathogen in community-acquired pneumonia in the region. This type of pneumonia develops in stroke patients with dysphagia, which can induce aspiration and aggravate the patient’s condition and further complicate their recovery [13].

In the early stages of the global COVID-19 outbreak, healthcare providers often resorted to an empirical antibiotic treatment of hospitalized patients, especially those infected with COVID-19. Unfortunately, this approach leads to antibiotic resistance [8]. This study suggested that bacterial strains were more diverse during the COVID-19 period, and bacteria developed resistance to various antimicrobial agents, including β-lactamase antibiotics for Gram-negative bacteria and methicillin for Gram-positive bacteria.

This study showed that fungal infections caused by *C. albicans* and *C. tropicalis* represent only a minority of the causes of infection in AIS. All *Candida* species were cultured from the sputum sample, whilst *Cryptococcus neoformans* was isolated from the cerebrospinal fluid. *Candida* species are naturally occurring microorganisms in the human body that are generally harmless and are often detected in sputum samples as uninvolved third parties. However, *Candida* infections, whether endogenous or from the hospital environment, are increasing in immunocompromised post-stroke patients. To address this problem and develop feasible solutions, molecular biology research is needed [23].

While the human microbiome does not usually contain *C. neoformans*, patients with symptomatic cryptococcosis usually have a well-defined immunocompromised status, including advanced cancers, DM, sarcoidosis, organ transplantation, HIV, and prolonged corticosteroid therapy. In this study, the AIS patient with Cryptococcosis has HIV as a condition that predisposes immunocompromised patients with a normal leukocyte count. However, the normal leukocyte count does not imply that the function of the leucocyte and its subsets was normal, as we did not have data on those. Based on the previous study, it is now estimated that approximately 20% of patients with Cryptococcosis do not have HIV infection [24].

Intensive and stroke unit care was associated with a decrease in microbiology culture positivity rates in AIS during the five years, both in the non-COVID-19 and COVID-19 periods. It has been shown that the rapid transfer of patients to an intensive care or stroke unit can improve functional outcomes, reduce the need for institutional care and disability, and reduce complications such as dysphagia screening and better management, also reducing mortality by more than 20% compared to regular ward care [25].

In the non-COVID-19 period, urinary catheterization was associated with an increase in pathogen detection, while antibiotic administration was associated with a decrease in the microbiological culture positivity rate. As expected, leukocytosis upon admission was associated with a higher microbiology culture positivity rate in the non-COVID-19 period. In contrast, leukocytosis and steroids were associated with lower pathogen detection in microbiological culture in the COVID-19 period. Leukocytes are recruited and activated to destroy the pathogen. During the COVID-19 period, viral infections dominated among patients. Therefore, the positivity rate of the microbiological culture was low. Cytokines produced by dendritic cells, macrophages, and other cell types during the innate immune response are involved in the leukocyte reactions. If the initial immune response has not cleared the viral infection, the administration of steroids is thought to downregulate immune-mediated lung injury and the cytokine storm. When properly administered, systemic steroids have been reported to increase the survival rate of COVID-19 patients and modulate the immune response, particularly in sepsis, by reversing septic shock [20].

TPN has been associated with a decrease in the positivity rate of microbiological culture in AIS in the COVID-19 period. TPN restores clinical development and the immunologic response against pathogens directly related to malnutrition. Malnutrition is associated with poorer outcomes, higher mortality, and poor prognosis in stroke patients. Patients with stroke often have dysphagia, which promotes malnutrition. During the COVID-19 period, it is recommended that patients consider starting tube feeding within 24 h of admission to the hospital [26,27]. However, in previous studies, TPN use can disrupt gut mucosal and epithelial barrier function, which might affect the gut immune response, for instance, gut microbiome shifting and the dysregulation of T-cells and cytokines. Therefore, there is a possibility of gaining access to blood vessels [28].

During the COVID-19 period, strokes were often more severe, and there was a greater need for early tracheostomy to prevent aspiration. Infection control procedures were heavily utilized, and healthcare workers were advised to use personal protective equipment and practice hand hygiene, as these measures can reduce infection and cross-contamination. In our study, tracheostomy was reduced by 20% of pathogen growth in microbiologic culture in AIS during the COVID-19 period. Though DSA was not associated with microbiology culture positivity rates in AIS during both the non-COVID-19 and COVID-19 periods, DSA was associated with a decrease in microbiology culture positivity rates in AIS of 22% over the five years, suggesting that DSA has been useful to identify the blockages in cerebral arteries and for direct treatment, enabling early recovery.

This study has several drawbacks. This study collected data from the first 7 days of AIS patients’ admission, without further follow-up until patients’ discharge. Hence, it remains unclear whether infections after AIS in this study influenced the length of hospital stay, as in a previous study [4]. Due to the retrospective design, some data could not be collected, for instance, the size or location of the brain lesion or detailed clinical and other examinations of the patient. In addition, the microbiologic culture results were bacteria and fungi but not anaerobic, atypical, or fastidious bacteria due to an equipment limitation. Because this study was conducted at a single center, the sample size was small. Even though this study was performed in a large neurology referral center, the results may not be generalizable to primary stroke units and other facilities. It is suggested that future studies need to determine the prevalence and risk factors associated with infections after AIS in different settings and address the long-term influence of infections in AIS. The prevalence and risk factors associated with infection after ischemic stroke subtypes, non-ischemic stroke, or subsets of acute stroke patients are all important and challenging issues and deserve to be evaluated in further studies.

## 5. Conclusions

This study highlighted the concern about infections in patients with AIS, an issue of significant public health importance. Positive microbiological culture revealed that Gram-negative bacteria such as *K. pneumoniae*, *E. coli*, and *A. baumannii* and Gram-positive bacteria such as *S. aureus* were responsible for infections in hospitalized patients with AIS, suggesting a possible nosocomial infection. During the COVID-19 period, the bacteria developed resistance to various antimicrobial agents, including β-lactamase antibiotics for Gram-negative bacteria and methicillin for Gram-positive bacteria. Intensive care and stroke unit stay resulted in a reduction in the microbiology culture positivity rate due to dysphagia screening and better management. Urinary catheter and leukocytosis were positively associated with pathogen deposition in microbiologic culture in the non-COVID-19 period, while steroids, TPN, and tracheostomy were negatively associated in the COVID-19 period.

## Figures and Tables

**Table 1:** Basic characteristics of the test subjects.

Characteristic	*n*	%
Age, mean ± SD, years	60.0 ± 5.0	
Age ≥ 60 years, *n* (%)	313	52.3
Male, *n* (%)	407	67.9
Intensive/stroke unit care, *n* (%)	214	35.7
DM, *n* (%)	163	27.2
HIV, *n* (%)	24	4.0
COVID-19, *n* (%)	310	51.8
Leukocytosis (>10,000/μL), *n* (%)	18	3.0
Leukopenia (<5000/μL), *n* (%)	392	65.4
Ventilator, *n* (%)	61	10.2
CVC, *n* (%)	179	29.9
NGT, *n* (%)	55	9.2
Urinary catheter, *n* (%)	31	5.2
Antibiotic, *n* (%)	367	61.3
Steroid, *n* (%)	55	9.2
TPN, *n* (%)	42	7.0
Transfusion, *n* (%)	33	5.5
Tracheostomy, *n* (%)	39	6.5
DSA, *n* (%)	10	1.7
Head surgery, *n* (%)	8	1.3

COVID: coronavirus disease; CVC: central venous catheter; DM: diabetes mellitus; DSA: digital subtraction angiography; HIV: human immunodeficiency virus; µL: microliter; NGT: nasogastric tube; SD: standard deviation; TPN: total parenteral nutrition; UTI: urinary tract infection.

**Table 2:** Clinical manifestation of the infection.

Characteristic	Non-COVID-19 Period		COVID-19 Period		*p*-Value
	*n*	%	*n*	%	
Fever	8	6.7	111	23.1	<0.001
Sepsis	34	28.6	189	39.4	<0.001
Pneumonia	39	32.8	199	41.5	<0.001
Meningitis/encephalitis	12	10.1	14	2.9	0.366
Ulcer	4	3.4	7	1.5	0.166
UTI	20	16.8	4	0.8	0.248

COVID: coronavirus disease, UTI: urinary tract infection.

**Table 3:** Positivity rate of the sample cultures.

Year	2018		2019		2020		2021		2022		In Total	
	*n*	%	*n*	%	*n*	%	*n*	%	*n*	%	*n*	%
Sample	42	100	18	100	171	100	309	100	59	100	599	100
LCS	2	4.8	3	16.7	7	4.1	7	2.3	7	11.9	26	4.3
Blood	2	4.8	2	11.1	137	80.1	263	85.1	19	32.2	423	70.6
Bronchial lavage	0	0.0	1	5.6	1	0.6	0	0.0	0	0.0	2	0.3
Sputum	37	88.1	11	61.1	11	61.1	28	9.1	10	16.9	111	18.5
Pleural effusion	0	0.0	1	5.6	0	0.0	1	0.3	0	0.0	1	0.2
Pus	1	2.4	0	0.0	0	0.0	7	2.3	3	5.1	11	1.8
Urine	0	0.0	1	5.6	1	0.6	3	1.0	19	32.2	24	4.0
Synovial fluid	0	0.0	0	0.0	0	0.0	0	0.0	1	1.7	1	0.2
Positivity rate	39	92.9	10	55.6	24	14.0	35	11.3	20	33.9	128	21.4
LCS	1	50.0	0	0.0	1	14.3	0	0.0	1	14.3	3	11.5
Blood	0	0.0	0	0.0	1	0.7	9	3.4	1	5.3	11	2.6
Bronchial lavage	0	0.0	1	100	1	100	0	0.0	0	0.0	2	0.0
Sputum	37	100	10	90.9	21	84.0	23	82.1	9	90.0	100	90.1
Pleural effusion	0	0.0	0	0.0	0	0.0	1	100	0	0.0	1	100
Pus	1	100	0	0.0	0	0.0	3	42.9	3	100	7	63.6
Urine	0	0.0	0	0.0	1	100	0	0.0	6	31.6	7	29.2
Synovial fluid	0	0.0	0	0.0	0	0.0	0	0.0	1	100	1	100

LCS: liquor cerebrospinal.

**Table 4:** Proportion of the pathogens.

	2018		2019		2020		2021		2022		Total	
	*n*	%	*n*	%	*n*	%	*n*	%	*n*	%	*n*	%
Annual prevalence	39	92.9	10	55.6	24	14.0	35	11.3	20	33.9	128.0	21.4
Proportion:												
Gram-negative	17	43.6	6	60.0	16	66.7	17	48.6	12	60.0	68	53.1
Gram-positive	17	43.6	2	20.0	5	20.8	13	37.1	3	15.0	40	31.3
Fungi	5	12.8	2	20.0	3	12.5	5	14.3	5	25.0	20	15.6

**Table 5:** Characteristics of the pathogens.

Characteristic	Non-COVID-19 Period		COVID-19 Period		*p*-Value
	*n*	%	*n*	%	
Gram-negative	35	11.4	33	6.9	<0.001
Gram-positive	22	18.5	18	3.8	<0.001
Fungi	12	10.1	8	1.7	<0.001

**Table 6:** Univariate analysis of factors associated with infection in AIS.

Characteristic		Total		Non-COVID-19 Period			COVID-19 Period		
	*n*	%	*p*-Value	*n*	%	*p*-Value	*n*	%	*p*-Value
	599	100	-	119	100	-	480	100	-
Age ≥ 60 years, *n* (%)	313	52.3	0.116	59	49.6	0.712	254	52.9	0.165
Male, *n* (%)	407	67.9	0.283	87	73.1	1.000	320	66.7	0.465
Intensive/stroke unit care, *n* (%)	214	35.7	<0.001	73	61.3	0.004	141	29.4	<0.001
DM, *n* (%)	163	27.2	0.350	22	18.5	<0.001	141	29.4	0.879
HIV, *n* (%)	24	4.0	0.013	2	1.7	0.509	22	4.6	0.003
COVID-19, *n* (%)	310	51.8	0.009	1	0.8	0.420	309	64.4	<0.001
Leukocytosis, *n* (%)	18	3.0	0.032	85	71.4	0.004	307	64.0	<0.001
Leukopenia, *n* (%)	392	65.4	0.929	4	3.4	0.130	14	2.9	0.235
Ventilator, *n* (%)	61	10.2	<0.001	18	15.1	0.075	43	9.0	<0.001
CVC, *n* (%)	179	29.9	<0.001	62	52.1	0.015	117	24.4	<0.001
NGT, *n* (%)	55	9.2	<0.001	23	19.1	0.817	32	6.7	0.096
Urinary catheter, *n* (%)	31	5.2	0.866	12	10.1	0.004	19	4.0	0.071
Antibiotic, *n* (%)	367	61.3	0.012	61	53.3	<0.001	306	63.7	0.313
Steroid, *n* (%)	55	9.2	<0.001	21	17.6	<0.001	34	7.1	0.025
TPN, *n* (%)	42	7.0	<0.001	19	16.0	0.448	23	4.8	0.001
Transfusion, *n* (%)	33	5.5	0.031	12	10.1	1.000	21	4.4	0.161
Tracheostomy, *n* (%)	39	6.5	<0.001	17	14.3	1.000	22	4.6	<0.001
DSA, *n* (%)	10	1.7	<0.001	7	5.9	0.021	3	0.6	1.000
Head surgery, *n* (%)	8	1.3	<0.001	1	0.8	1.000	7	1.5	<0.001

COVID: coronavirus disease; CVC: central venous catheter; DM: diabetes mellitus; DSA: digital subtraction angiography; HIV: human immunodeficiency virus; µL: microliter; NGT: nasogastric tube; TPN: total parenteral nutrition; UTI: urinary tract infection.

**Table 7:** Multivariate analysis of factors associated with infections after AIS.

Factors		Total		Non-COVID-19 Period			COVID-19 Period		
	OR	95% CI	*p*-Value	OR	95% CI	*p*-Value	OR	95% CI	*p*-Value
Intensive/stroke unit care	0.23	0.14–0.37	<0.001	0.35	0.14–0.89	<0.001	0.17	0.09–0.33	<0.001
COVID-19	4.66	2.69–8.08	<0.001	-	-	-	-	-	-
Leukocytosis	-	-	-	5.51	1.80–16.91	0.003	0.32	0.13–0.76	0.010
Urinary catheter	-	-	-	13.12	1.68–102.1	0.014	-	-	-
NGT	0.46	0.23–0.92	0.029	-	-	-	-	-	-
Antibiotic	-	-	-	0.15	0.06–0.37	<0.001	-	-	-
Steroid	0.23	0.12–0.47	<0.001	-	-	-	0.33	0.13–0.87	0.024
TPN	0.46	0.22–0.99	0.045	-	-	-	0.21	0.07–0.57	0.002
Tracheostomy	-	-	-	-	-	-	0.20	0.08–0.51	0.001
DSA	0.22	0.05–1.00	0.050	-	-	-	-	-	-

AIS: acute ischemic stroke; COVID-19: coronavirus disease; DSA: digital subtraction angiography; NGT: nasogastric tube; TPN: total parenteral nutrition.

## Data Availability

The data are available to researchers who can obtain the data upon reasonable request or attempt to duplicate the process or results by contacting the corresponding author directly.

## Supplementary Materials

The following supporting information can be downloaded at: https://www.mdpi.com/article/10.3390/epidemiologia6030046/s1, Table S1: Multivariate analysis of factors associ- ated with infection after AIS; Table S2: Multivari- ate analysis of factors associated with infection af- ter AIS in non-COVID-19 period; Table S3: Multi- variate analysis of factors associated with infec- tion after AIS in COVID-19 period.

## Abbreviations

The following abbreviations are used in this manuscript:

AIS	Acute ischemic stroke
CFU	Colony-forming unit
CI	Confidence interval
COPD	Chronic obstructive pulmonary disease
COVID-19	Coronavirus disease 2019
CVC	Central venous catheter
CVD	Cerebrovascular disease
DM	Diabetes mellitus
DSA	Digital subtraction angiography
HCU	Intensive care unit
HIV	Human immunodeficiency virus
ICU	Intensive care unit
LCS	liquor cerebrospinal
µL	Microliter
NBC	National Brain Center Hospital
NGT	Nasogastric tube
NIHSS	National Institutes of Health Stroke Scale
OR	Odds ratio
SCU	Stroke Care Unit
SD	Standard deviation
TPN	Total parenteral nutrition
UTI	Urinary tract infection
WHO	World Health Organization
