# Supplementary material for: Prevalence and Factors Associated with Infections After Acute Ischemic Stroke: A Single-Center Retrospective Study over Five Years
Source: Epidemiologia (Basel). 2025 Aug 11;6(3):46. doi: 10.3390/epidemiologia6030046 (PMC12372096; doi:10.3390/epidemiologia6030046)
Supplement: Supplementary file 1 [file epidemiologia-06-00046-s001.zip › epidemiologia-3709160-supplementary.pdf]

Table S1. Multivariate analysis of factors associated with infection after AIS

Variable	Initial model			Final model		
	**OR**	**95%CI**	**p-value**	**OR**	**95%CI**	**p-value**
Age ≥ 60 years	0.769	0.468 – 1.264	0.300	-	-	-
Intensive/stroke unit care	0.246	0.141 – 0.430	< 0.001	0.226	0.137 - 0.374	< 0.001
HIV	0.428	0.147 – 1.250	0.121	-	-	-
COVID-19	4.652	2.645 – 8.184	< 0.001	4.661	2.688 - 8.083	< 0.001
Leukocytosis	0.970	0.564 – 1.667	0.911	-	-	-
Ventilator	0.961	0.413 – 2.238	0.926	-	-	-
CVC	1.066	0.606 – 1.875	0.826	-	-	-
NGT	0.424	0.209 - 0.862	0.018	0.462	0.231 - 0.923	0.029
Antibiotic	0.770	0.454 – 1.307	0.334	-	-	-
Steroid	0.294	0.133 - 0.649	0.002	0.234	0.117 - 0.467	< 0.001
TPN	0.529	0.213 - 1.317	0.171	-	-	-
Transfusion	1.134	0.456 - 2.819	0.787	-	-	-
Tracheostomy	0.506	0.232 - 1.102	0.086	0.464	0.219 - 0.985	0.045
DSA	0.195	0.039 - 0.967	0.045	0.216	0.047 - 1.002	0.050
Head surgery	0.300	0.056 - 1.610	0.160	-	-	-

Table S2. Multivariate analysis of factors associated with infection after AIS in non-COVID-19 period

Variable	Initial model			Final model		
	**OR**	**95%CI**	**p-value**	**OR**	**95%CI**	**p-value**
Intensive care	0.281	0.093 – 0.845	0.024*	0.346	0.135 – 0.887	< 0.001*
DM	0.153	0.015 – 1.525	0.109	-	-	-
Leukocytosis	4.967	1.487 – 16.593	0.009*	5.514	1.798 – 16.908	0.003*
Leukopenia	-	-	0.999	-	-	-
Ventilator	3.142	0.475 – 20.796	-	-	-	-
CVC	1.457	0.480 – 4.420	-	-	-	-
Urinary catheter	6.030	0.893 – 40.707	0.065	13.117	1.684 – 102.184	0.014*
Antibiotic	0.249	0.079 – 0.784	0.018*	0.151	0.059 – 0.368	< 0.001*
Steroid	0.192	0.019 – 1.921	0.192	-	-	-
DSA	-	-	0.999	-	-	-

Table S3. Multivariate analysis of factors associated with infection after AIS in COVID-19 period

Variable	Initial model			Final model		
	OR	95%CI	p-value	OR	95%CI	p-value
Age ≥ 60 years	0.705	0.356 – 1.394	0.315	-	-	-
Intensive care	0.296	0.132 – 0.662	0.003	0.170	0.086 – 0.334	< 0.001*
HIV	1.150	0.973 – 1.358	0.101	-	-	-
COVID-19	0.925	0.849 – 1.008	0.076	-	-	-
Leukocytosis	0.374	0.149 – 0.937	0.036	0.318	0.133 – 0.762	0.010*
Leukopenia	-	-	0.999	-	-	-
Ventilator	0.864	0.276 – 2.705	0.802	-	-	-
CVC	0.604	0.286 – 1.274	0.185	-	-	-
NGT	1.091	0.944 – 1.261	0.239	-	-	-
Urinary catheter	0.713	0.179 – 0.179	0.632	-	-	-
Steroid	0.364	0.123 – 1.081	0.069	0.332	0.127 – 0.866	0.024*
TPN	1.153	0.975 – 1.362	0.096	0.205	0.073 – 0.572	0.002*
Transfusion	0.966	0.818 – 1.141	0.683	-	-	-
Tracheostomy	0.234	0.084 – 0.651	0.005	0.195	0.075 – 0.510	0.001*
Head surgery	1.176	0.938 – 1.475	0.160	-	-	-

* $p < 0.05$ indicates statistically significant related variables. COVID: coronavirus disease, CVC: central venous catheter; DM: diabetes mellitus; DSA: digital subtraction angiography; HIV: human immunodeficiency virus; μL: microliter; NGT: nasogastric tube; TPN: total parenteral nutrition; UTI: urinary tract infection.
